# Safety and Efficacy of OnabotulinumtoxinA for Treatment of Crow’s Feet Lines in Chinese Subjects

**DOI:** 10.1097/GOX.0000000000002079

**Published:** 2019-01-22

**Authors:** Yan Wu, Gang Wang, Chengxin Li, Cheri Mao, Xiaofang Lei, Elisabeth Lee

**Affiliations:** From the * Peking University First Hospital, Beijing, China; † Xijing Hospital, The Fourth Military Medical University, Shaanxi Province, China; ‡ Chinese PLA General Hospital, Beijing, China; § Allergan plc, Irvine, Calif.

## Abstract

**Background:**

This study evaluated the safety and efficacy of onabotulinumtoxinA for the treatment of crow’s feet lines (CFL) in Chinese subjects.

**Methods:**

This 5-month, double-blind, randomized, parallel-group, placebo-controlled phase 3 study was conducted in China. Subjects with moderate-to-severe CFL at maximum smile received a single treatment of onabotulinumtoxinA 24 U (total; n = 316) or placebo (n = 101) on day 1. The primary efficacy measure was the proportion of investigator-assessed responders (achieved CFL severity of none or mild at maximum smile using the Facial Wrinkle Scale with Asian Photonumeric Guide at day 30). Additional endpoints included other response definitions (achieving at least 1-grade improvement at maximum smile and at rest using the Facial Wrinkle Scale with Asian Photonumeric Guide at day 30), duration of effect, subject-reported outcomes, and safety.

**Results:**

All efficacy endpoints were met. At day 30, the proportion of subjects achieving none or mild severity at maximum smile was significantly greater (*P* < 0.001) in the onabotulinumtoxinA group (63.9%) versus the placebo group (5.0%). The proportion of subjects assessing the change in CFL appearance as much improved/very much improved was also significantly greater with onabotulinumtoxinA than placebo (*P* < 0.001). Subjects’ self-assessed outcomes were similar to investigator-assessed results. Median duration of effect with onabotulinumtoxinA was ≥5 months using all responder definitions. A low occurrence of treatment-related adverse events was reported, with no new safety findings.

**Conclusions:**

OnabotulinumtoxinA 24 U was effective and well tolerated for the treatment of CFL in Chinese subjects, with responses maintained over 5 months.

## Abstract 2

Supplemental Digital Content is available in the text.

## INTRODUCTION

The use of botulinum toxin injections for minimally invasive facial aesthetic procedures has become common worldwide, with more than 4.6 million procedures performed in 2015.^1^ OnabotulinumtoxinA (Botox Cosmetic; Allergan plc, Dublin, Ireland) is approved for the treatment of glabellar lines in more than 75 countries and crow’s feet lines (CFL) in more than 55 countries.

Initial studies demonstrating the effectiveness of onabotulinumtoxinA for the treatment of CFL were conducted in predominantly Caucasian populations.^2,3^ These trials used the Facial Wrinkle Scale (FWS; Allergan plc, Dublin, Ireland) to evaluate CFL severity before and after treatment.^2,3^ Patterns of CFL morphology may vary across Asian and Caucasian populations.^4,5^ Therefore, a modified version of the FWS scale with an Asian Photonumeric Guide (FWS-A) was developed and validated for assessing the severity of upper facial lines in Asian subjects, including CFL. This scale has been successfully used to assess the efficacy of onabotulinumtoxinA for treatment of CFL in Japanese subjects.^6^

The present study evaluated the safety, efficacy, and patient-reported outcomes found with onabotulinumtoxinA for the treatment of moderate-to-severe CFL in Chinese subjects.

## METHODS

### Study Design

This 5-month, multicenter, double-blind, randomized, parallel-group, placebo-controlled phase 3 study was conducted at 9 sites in China from September 2014 through June 2015 (clinicaltrials.gov identifier: NCT02195687). Subjects were randomized in a 3:1 ratio to receive a single treatment of onabotulinumtoxinA 24 U or placebo on day 1 (**see figure, Supplemental Digital Content 1**, which displays the study design, http://links.lww.com/PRSGO/A962). At study entry, subjects were assigned a randomization number, and an interactive voice response system was used to manage the randomization and treatment assignment based on a randomization scheme prepared by Allergan Biostatistics. Investigators and subjects were blinded to the treatment administered.

A properly constituted Independent Ethics Committee approved the study protocol before initiation of the study at each site. The study was conducted in compliance with China’s Good Clinical Practice guidelines and with the International Council for Harmonisation guidelines for Good Clinical Practice, topic E6. Written informed consent and privacy-related documentation, in compliance with Chinese and local privacy requirements, were obtained from each subject before enrollment.

## SUBJECTS

This study enrolled Chinese females and males aged at least 18 years with moderate-to-severe bilateral CFL at maximum smile (same grade on both sides), as assessed by both the investigator and the subject using the FWS-A. Key exclusion criteria were concurrent treatment with botulinum toxin or treatment within 1 year before enrollment; known immunization or hypersensitivity to botulinum toxin; any medical condition that may result in increased subject risk; pregnancy; previous surgical procedures at the treatment sites; eyebrow or eyelid ptosis; and eyelid hooding or other skin laxity unlikely to benefit from onabotulinumtoxinA treatment or likely to interfere with CFL assessments.

### Treatment

**Fig. 1.**
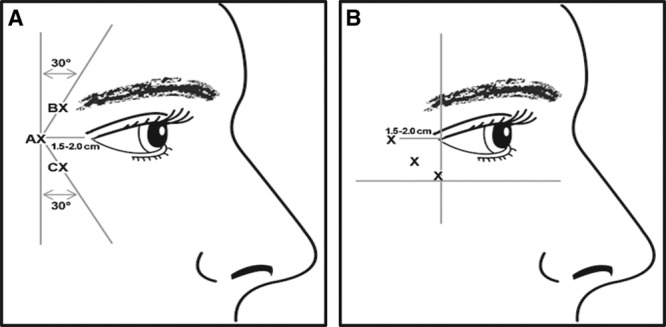
Injection pattern and allowable modification for the treatment of CFL. CFL injection pattern (A). The first injection (line AX) was in the orbicularis oculi at the level of the lateral canthus at least 1.5–2.0 cm temporal to the lateral canthus and just temporal to the lateral orbital rim. The second injection (line BX) was 1.0–1.5 cm above this first injection site and at an approximate 30° angle medially. The final injection (line CX) was made 1.0–1.5 cm below the first injection at an approximate 30° angle medially. Modified CFL injection pattern (B). If the lines in the lateral canthal areas were positioned primarily below the lateral canthus, injections could be angled from anteroinferior to superposterior, provided the most anterior injection was made lateral to a line drawn vertically from the lateral canthus and the most inferior injection was placed superior to the maxillary prominence. Reprinted with permission from Carruthers A, *et al. Dermatol Surg* 2014; **40**: 1181–1190.^2^

Treatment consisted of 6 intramuscular injections of 0.1 mL each (3 injections per side) of onabotulinumtoxinA (4 U per injection site; total dose, 24 U) or placebo (sterile 0.9% sodium chloride without preservative). The 2 treatment patterns available to the investigators, who selected the treatment pattern based on the positioning of the subject’s CFL, are shown in Figure 1.^2^ Injections were administered with the needle tip bevel up and oriented away from the eye.

### Assessments

#### Efficacy Measures

The primary efficacy measure was the percentage of subjects who were responders, with responders defined as those achieving investigator-rated CFL severity of none or mild at maximum smile on the FWS-A (0 = none, 1 = mild, 2 = moderate, and 3 = severe). Day 30 after treatment was the primary time point. To ensure consistent rating of CFL severity, investigators at each center were trained in rating CFL severity using the FWS-A before enrolling subjects. Two validated versions of the FWS-A were provided: an investigator version with investigator-oriented instructions in Chinese and a subject version with subject-oriented instructions in Chinese. Each investigator and subject rated CFL severity at the same time, using their own FWS-A. The ratings were made first at rest, then at maximum smile. Subjects and investigators were not permitted to discuss the results of the FWS-A assessment with each other.

Additional efficacy measures based on FWS-A scores included the following: the proportion of subjects achieving at least a 1-grade improvement in CFL severity at maximum smile and at rest at day 30, and Kaplan-Meier estimated duration of effect. The duration of effect was defined as the median time until loss of efficacy (from responder to nonresponder status for day 30 responders), using 3 different FWS-A responder definitions (CFL severity of none or mild at maximum smile, ≥1-grade improvement in CFL severity at maximum smile, and ≥1-grade improvement in CFL severity at rest) for both the investigator- and subject-rated FWS-A scores.

#### Patient-reported Outcome Measures

The Subject’s Global Assessment of Change in CFL (SGA-CFL), a single-item measure, asked subjects to assess their CFL appearance at the current time compared with their CFL appearance before treatment on a 7-point scale ranging from 1 (very much improved) to 7 (very much worse). On the Self-Perception of Age (SPA) questionnaire, a single-item measure, subjects indicated looking their current age, looking older than their current age (recording the number of years older they appear), or looking younger than their current age (recording number of years younger they appear).^7^ The Subject Assessment of Satisfaction with Appearance, a single-item measure, was used to assess each subject’s satisfaction with their CFL appearance at the current time on a 5-point scale, ranging from 1 (very unsatisfied) to 5 (very satisfied). Subject satisfaction with treatment was assessed by the Facial Line Satisfaction Questionnaire (FLSQ) overall satisfaction item, which is rated on a 5-point Likert scale ranging from −2 (very dissatisfied) to +2 (very satisfied).^8^ Subject satisfaction with their current CFL appearance was also assessed using the 11-item Facial Line Outcomes (FLO-11) questionnaire.^9^ The total score was calculated as the sum of the absolute means of the individual scores and was transformed to a scale from 0 (worst) to 100 (best), as previously described.^10^

### Safety

Safety measures included monitoring adverse events (AEs), neurologic assessments (focused symptoms questionnaire and focused neurologic examination), and vital signs.

### Statistical Analyses

All efficacy analyses were performed on the modified intent-to-treat population, which comprised all randomized subjects who received injection of study drug. Safety analyses were based on all subjects who received injection of study drug and were analyzed by actual treatment received, regardless of randomization. For all responder analyses, between-group *P* values were based on Cochran-Mantel-Haenszel tests stratified by baseline CFL severity at maximum smile using the investigator-rated FWS-A score. Kaplan-Meier survival methods were used to calculate the median duration of effect for FWS-A responders.

## RESULTS

### Subjects

Of 419 randomized subjects, 417 received treatment and were included in the modified intent-to-treat population (n = 316, onabotulinumtoxinA; n = 101, placebo). Most subjects completed the study, including 98.4% (n = 311) of subjects in the onabotulinumtoxinA group and 98.0% of (n = 99) subjects in the placebo group. Reasons for discontinuation in both groups were limited to lost to follow-up (n = 4, onabotulinumtoxinA; n = 1, placebo) and personal reasons (n = 1, onabotulinumtoxinA; n = 1, placebo).

Overall, 86.3% of subjects were female and the mean age was 46.4 years (Table 1). Baseline FWS-A CFL severity at maximum smile was assessed by the investigator as moderate in 48.4% of subjects and as severe in 51.6%. Demographic characteristics and baseline FWS-A CFL severity were similar between the treatment groups.

**Table 1.**
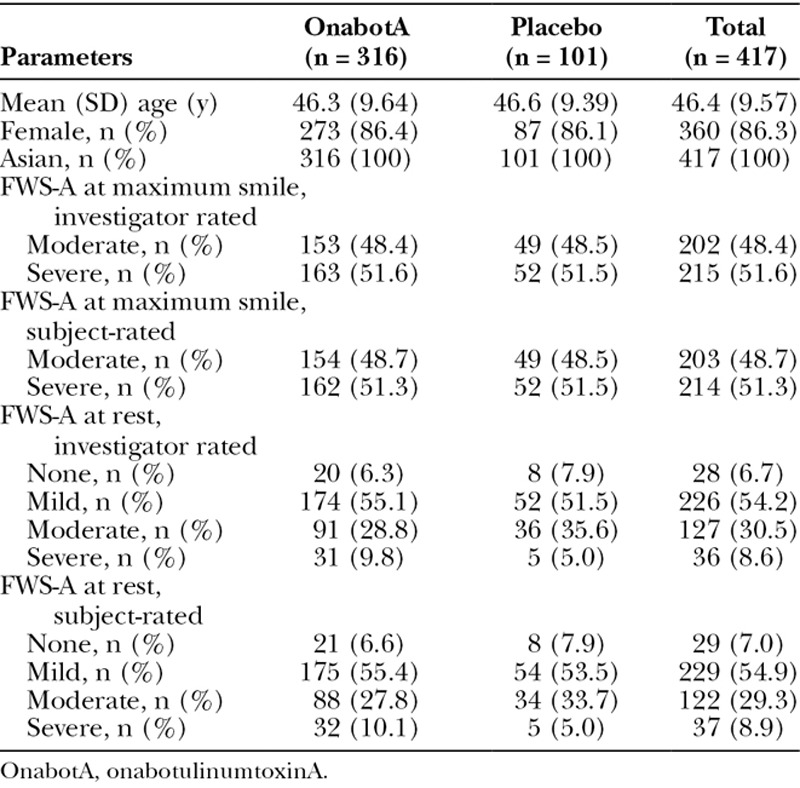
Demographics and Baseline Characteristics

### Efficacy

#### Responder Rates Based on Investigator-rated CFL Severity

**Fig. 2.**
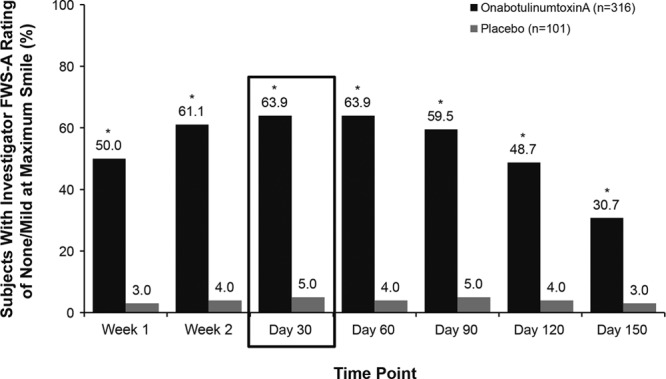
Proportion of subjects who achieved a rating of none or mild at maximum smile based on investigator’s FWS-A Photonumeric Guide rating of the severity of CFL (modified intent-to-treat population). *P < 0.001 for onabotulinumtoxinA versus placebo based on Cochran-Mantel-Haenszel tests, stratified by baseline severity of CFL at maximum smile.

The primary efficacy endpoint of this study was met. The proportion of subjects who were responders, defined as those achieving investigator-rated CFL severity of none or mild at maximum smile at day 30 (primary efficacy time point), was significantly greater (*P* < 0.001) in the onabotulinumtoxinA group [63.9% (202/316)] versus the placebo group [5.0% (5/101)]. The proportion of responders was significantly greater with onabotulinumtoxinA compared with placebo at all other time points assessed in this 5-month study (*P* < 0.001; Fig. 2).

**Fig. 3.**
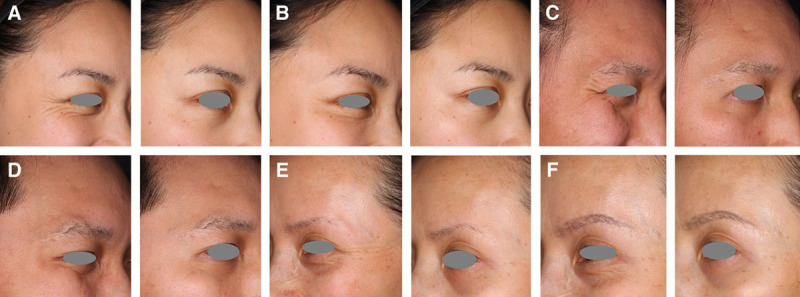
Investigator-rated severity of CFL is shown in representative subjects at maximum smile (left) and at rest (right) before treatment and at day 30 following treatment with onabotulinumtoxinA. A 33-year-old female with a baseline FWS-A score of 2 at maximum smile (A) had a score of 0 at day 30 (B). A 31-year-old male with a baseline FWS-A score of 2 at maximum smile (C) had a score of 0 at day 30 (D). A 48-year-old female with a baseline FWS-A score of 3 at maximum smile (E) had a score of 0 at day 30 (F). FWS-A scores indicated CFL severity: 0 = none, 1 = mild, 2 = moderate, and 3 = severe.

For the purpose of illustration, Figure 3 shows representative subjects with moderate-to-severe CFL at maximum smile at baseline (day 1) who achieved an FWS-A score of none or mild at day 30, as determined by the investigator.

**Fig. 4.**
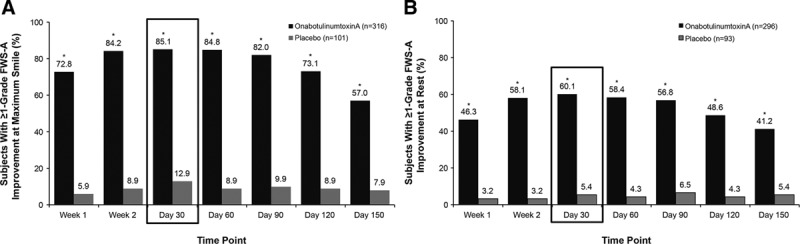
Proportion of subjects achieving an improvement from baseline of at least 1 grade in severity of CFL (A) at maximum smile and (B) at rest, based on investigator ratings on the FWS-A Photonumeric Guide. *P < 0.001 for onabotulinumtoxinA versus placebo.

The proportion of subjects with a response of at least a 1-grade improvement in investigator-rated FWS-A score at maximum smile was significantly greater in the onabotulinumtoxinA group compared with the placebo group at all time points (*P* < 0.001; Fig. 4A), with 85.1% (269/316) of subjects in the onabotulinumtoxinA group at the primary time point (day 30) compared with 12.9% (13/101) of subjects in the placebo group. The proportion of subjects with at least a 1-grade improvement in investigator-rated CFL severity at rest (among subjects with CFL rated at least mild at baseline) was significantly greater (*P* < 0.001) in the onabotulinumtoxinA group compared with the placebo group at all time points (Fig. 4B) at day 30 (60.1% [178/296] versus 5.4% [5/93], respectively).

#### Responder Rates Based on Subject-rated CFL Severity

The proportion of subjects who were responders based on the subject-rated FWS-A was similar to the proportion in the investigator-rated FWS-A at day 30, both at rest and at maximum smile, across all 3 responder definitions. At day 30, 60.8% (192/316) of subjects in the onabotulinumtoxinA group compared with 5.9% (6/101) of subjects in the placebo group rated their CFL severity at maximum smile as none or mild, a significantly greater response in the onabotulinumtoxinA group than in the placebo group (*P* < 0.001), which was maintained at all time points. Both at maximum smile and at rest, the proportions of subjects achieving improvement from baseline of at least 1 grade in subject-rated FWS-A scores were significantly greater *(P* < 0.001) in the onabotulinumtoxinA group compared with the placebo group at all time points.

### Duration of Effect

**Fig. 5.**
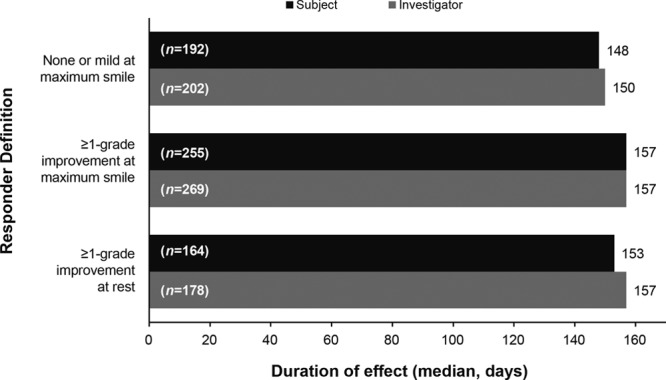
Duration of effect of onabotulinumtoxinA treatment in Chinese subjects, estimated using the Kaplan–Meier method. The n values within bars are number of responders at day 30.

The median duration of effect (time from response to loss of response) was at least 5 months for all FWS-A-based response definitions (Fig. 5). Median duration of effect across all responder definitions at maximum smile ranged from 150 to 157 days per investigator assessments and 148–157 days per subject assessments.

## SGA-CFL

Subjects assessed the change in their CFL appearance using the 7-point SGA-CFL scale with ratings ranging from “very much improved” to “very much worse” since starting treatment. The proportion of subjects who rated themselves as “very much improved” or “much improved” in the appearance of their CFL was significantly greater for the onabotulinumtoxinA group [54.4% (172/316)] than the placebo group [3.0% (3/101)] at day 30 and at all other time points (*P* < 0.001).

## SPA

Subjects were asked to assess the relationship between their CFL appearance and their age since starting treatment using 1 of 3 responses (“looking their current age,” “looking older than their current age,” and “looking younger than their current age”). The proportion of subjects who rated themselves as “looking their current age or older” at baseline and in a younger age category after treatment was significantly greater (*P* < 0.001) in the onabotulinumtoxinA group versus the placebo group at all time points (**see figure, Supplemental Digital Content 2**, which displays the subject’s SPA responder rates. Responders were defined as subjects who rated themselves as “looking their current age or older” at baseline and in a younger age category after treatment, http://links.lww.com/PRSGO/A963). At day 30, 53.9% (160/297) of subjects in the onabotulinumtoxinA group and 14.7% (14/95) of subjects in the placebo group rated themselves in a younger age category than at baseline (*P* < 0.001). At day 30, subject-perceived age was, on average, 2.7 years younger than baseline in the onabotulinumtoxinA group compared with 0.5 years younger in the placebo group (*P* < 0.001).

### FLSQ Overall Satisfaction

Subjects were asked to assess their overall satisfaction with treatment for correcting CFL lines using a 5-point scale with ratings ranging from “very unsatisfied” to “very satisfied.” The proportion of subjects who reported being “very satisfied” or “mostly satisfied” with treatment effect on the FLSQ overall satisfaction item was significantly greater for the onabotulinumtoxinA group (70.8% [221/312]) versus the placebo group (16.0% [16/100]) at day 30 and at all other time points (*P* < 0.001).

### Subject Satisfaction with Appearance of CFL

Subjects were asked to assess their satisfaction with their facial appearance due to CFL using a 5-point scale with ratings ranging from “very unsatisfied” to “very satisfied.” At baseline, most subjects (98.6% [411/417]) were neutral, unsatisfied, or very unsatisfied with their CFL appearance. The proportion of these subjects who reported being very satisfied or satisfied with CFL appearance at day 30 was significantly greater in the onabotulinumtoxinA group (57.7% [180/312]) than in the placebo group (4.0% [4/99]; *P* < 0.001 for day 30 and all other time points).

### FLO-11 Total Satisfaction with Appearance of CFL

Subjects were asked to assess their total satisfaction with their CFL appearance across all 11 items of the FLO-11, each of which was scored as 0 (not at all) to 10 (very much). The total score represents the sum of the individual item scores, transformed to a scale ranging from 0 (worst) to 100 (best).The mean ± SD total FLO-11 score was similar at baseline in both the onabotulinumtoxinA and placebo groups (38.0 ± 18.0 versus 36.2 ± 18.9; *P* = 0.395). By day 30, the mean change from baseline in FLO-11 scores was significantly greater for the onabotulinumtoxinA group than the placebo group (25.2 ± 21.5 versus 6.1 ± 15.3; *P* < 0.001), whereby higher scores indicate improvement in appearance-related outcomes.

### Safety

The proportion of subjects with treatment-emergent AEs (TEAEs) was similar in the onabotulinumtoxinA (7.6% [24/316]) and placebo (5.0% [5/101]) groups. Upper respiratory tract infection (1.3% [4/316] and 0.0% [0/101]) and injection site bruising (1.3% [4/316] and 3.0% [3/101]) were the most frequently reported TEAEs in the onabotulinumtoxinA and placebo groups, respectively (Table 2). All TEAEs were mild (75.9% [22/29]) or moderate ([20.7% [6/29]) in severity, except for 1 event of eye injury that was severe and was considered by the investigator to be unrelated to treatment. Treatment-related AEs occurred in 3 onabotulinumtoxinA–treated subjects (dizziness, 0.6% [2/316] and headache, 0.3% [1/316]). All of these events occurred soon after dosing, were mild i severity, and resolved completely within 3 days.

**Table 2.**
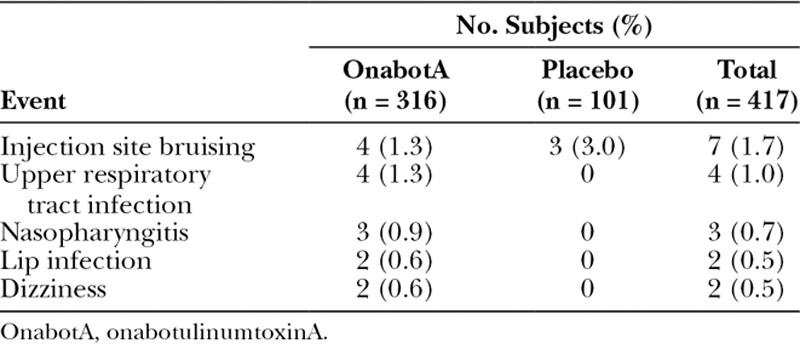
Treatment-emergent AEs Reported in More Than 1 Subject in Any Treatment Group

No subject discontinued the study due to a TEAE. No deaths, other serious TEAEs, possible spread of toxin events, neurologic events, or clinically meaningful changes in vital signs were reported.

## DISCUSSION

This pivotal phase 3 trial of onabotulinumtoxinA in Chinese subjects showed that a single treatment with onabotulinumtoxinA 24 U was effective for reducing the severity of moderate-to-severe CFL for at least 5 months. At day 30, the primary time point following treatment, statistically significant improvements in CFL severity favoring onabotulinumtoxinA over placebo were observed for all responder definitions as assessed by the investigator and by the subject. These results were consistent with subject-reported data: subject satisfaction with appearance and subject satisfaction with study drug treatment significantly improved in onabotulinumtoxinA–treated subjects versus placebo subjects.

The efficacy profile of onabotulinumtoxinA for the treatment of CFL in the current study in Chinese subjects is consistent with that reported in other studies that enrolled predominantly white subjects.^2,11^ The proportion of subjects who achieved investigator-rated CFL severity of none or mild at maximum smile with onabotulinumtoxinA 24 U at day 30 in the current study (63.9%) is consistent with the proportion observed in a similarly designed phase 3 study of predominantly white subjects (66.7%; n = 222, onabotulinumtoxinA 24 U group).^2^ The median duration of effect based on the responder definition of none or mild in CFL severity at maximum smile in the present study (150 days) was longer than a previous study in predominantly white subjects (118 days),^2^ providing further support for differences of CFL morphology between Asian and Caucasian populations.^4,5^

In this study, onabotulinumtoxinA–treated Chinese subjects consistently rated themselves as looking younger and reported significant improvements in satisfaction with CFL appearance and satisfaction with treatment versus placebo at all time points.^6^ These observations are similar to the findings of previous studies in predominantly white patient populations showing the positive effects of treatment of CFL with onabotulinumtoxinA on measures of perception of age, satisfaction with appearance, and satisfaction with treatment.^7,11,12^ Similar patient-reported outcomes have been reported for Japanese subjects treated with onabotulinumtoxinA in CFL.^6^

Treatment of CFL with onabotulinumtoxinA 24 U was safe and well tolerated, with no new safety findings observed. There were few treatment-related TEAEs, and no deaths, other serious TEAES, discontinuations because of AEs, possible spread of toxin events, or neurologic events were reported. The safety profile was consistent with other studies of onabotulinumtoxinA for the treatment of CFL and glabellar lines in non-Asian^2^ and Asian populations.^13,14^ Reasons for the longer duration of effect in Chinese subjects reported in this study versus previous CFL registration studies are likely multifactorial; further studies are warranted.

In conclusion, results indicate that for Chinese subjects, treatment with onabotulinumtoxinA 24 U is effective and well tolerated for reducing CFL severity at maximum smile and at rest, based on investigator and subject assessments. The median duration of response in this population was at least 5 months, and no new safety findings were identified.

## Supplementary Material
