# Supplementary figures and images for: Safety and Efficacy of OnabotulinumtoxinA for Treatment of Crow’s Feet Lines in Chinese Subjects
Source: Plast Reconstr Surg Glob Open. 2019 Jan 22;7(1):e2079. doi: 10.1097/GOX.0000000000002079 (PMC6382241; doi:10.1097/GOX.0000000000002079)

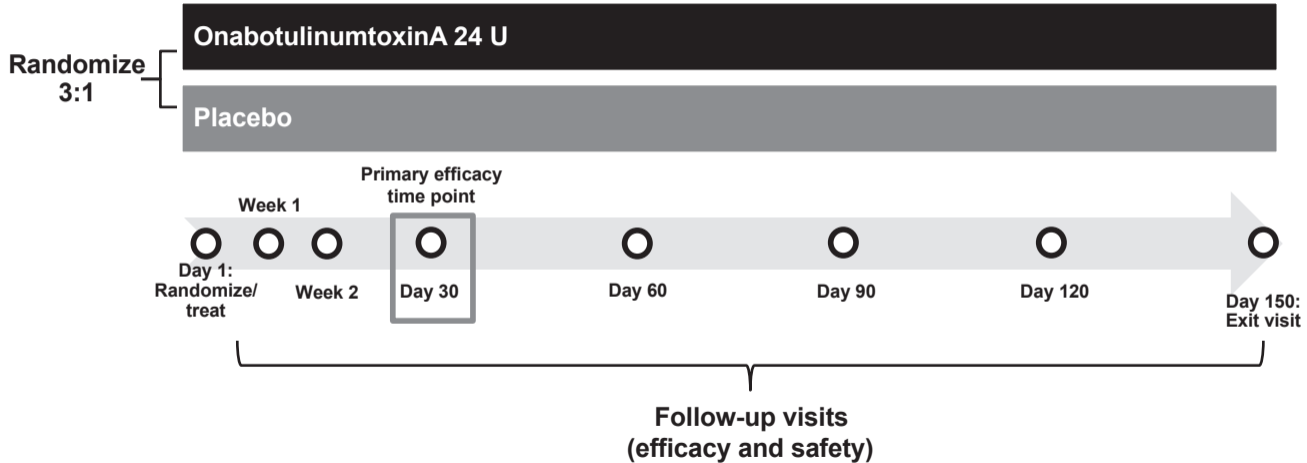

Supplement: Supplementary file 1 [file gox-7-e2079-s001.pdf]

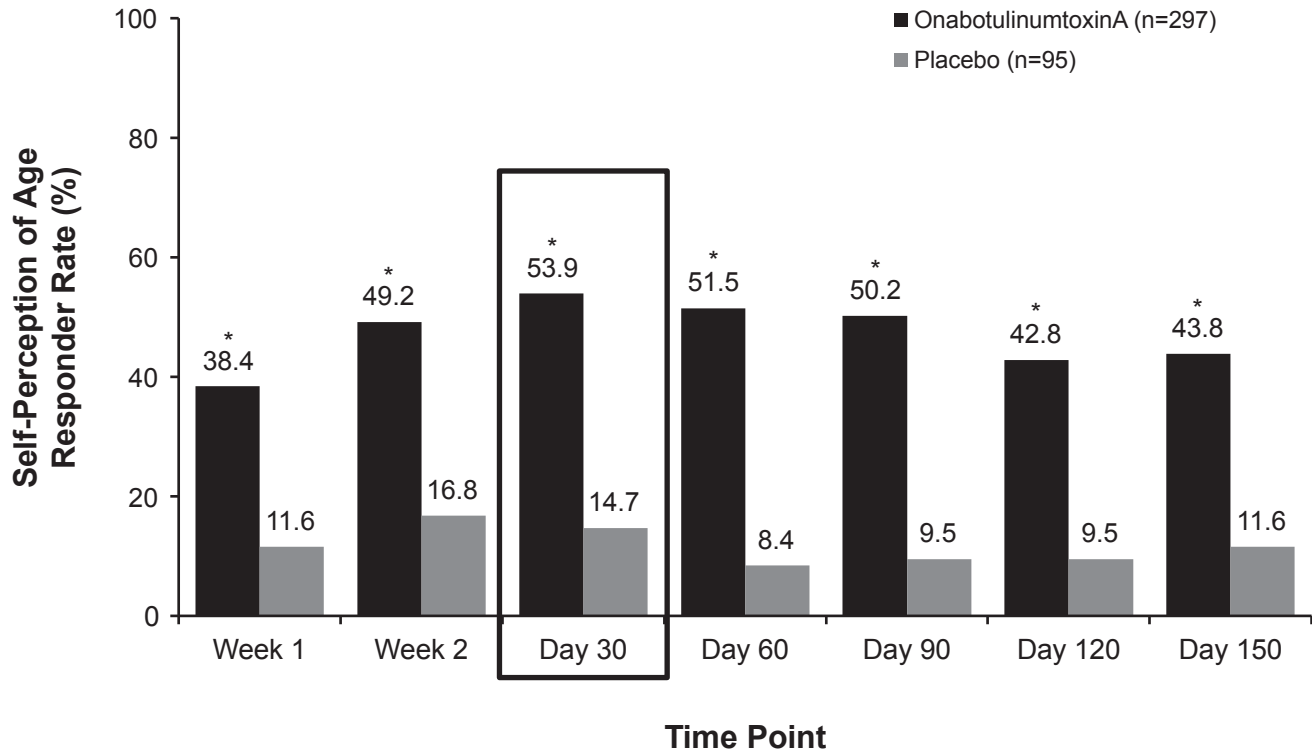

Supplement: Supplementary file 2 [file gox-7-e2079-s002.pdf]
